# Assessment of Tricuspid Regurgitation by Cardiac Magnetic Resonance Imaging: Current Role and Future Applications

**DOI:** 10.3390/jcm13154481

**Published:** 2024-07-31

**Authors:** Lobke L. Pype, Blanca Domenech-Ximenos, Bernard P. Paelinck, Nicole Sturkenboom, Caroline M. Van De Heyning

**Affiliations:** 1 Department of Cardiology, University Hospital Antwerp, 2650 Edegem, Belgium; lobke.pype@uza.be (L.L.P.);; 2 GENCOR Research Group, University of Antwerp, 2000 Antwerp, Belgium; 3 Department of Radiology, Hospital Clínic Barcelona, 08036 Barcelona, Spain; 4 Department of Cardiovascular Imaging, School of Biomedical Engineering & Imaging Sciences, King’s College London, London WC2R 2LS, UK; 5 Department of Cardiac Surgery, University Hospital Antwerp, 2650 Edegem, Belgium

**Keywords:** tricuspid regurgitation, cardiac magnetic resonance imaging, tricuspid valve

## Abstract

Tricuspid regurgitation (TR) is a prevalent valvular disease with a significant mortality rate. The evaluation of TR severity and associated right heart remodeling and dysfunction is crucial to determine the optimal therapeutic strategy and to improve prognosis. While echocardiography remains the first-line imaging technique to evaluate TR, it has many limitations, both operator- and patient-related. Cardiovascular magnetic resonance imaging (CMR) has emerged as an innovative and comprehensive non-invasive cardiac imaging technique with additional value beyond routine echocardiographic assessment. Besides its established role as the gold standard for the evaluation of cardiac volumes, CMR can add important insights with regard to valvular anatomy and function. Accurate quantification of TR severity, including calculation of regurgitant volume and fraction, can be performed using either the well-known indirect volumetric method or novel 4D flow imaging. In addition, CMR can be used to assess the impact on the right heart, including right heart remodeling, function and tissue characterization. Several CMR-derived parameters have been associated with outcome, highlighting the importance of multi-modality imaging in patients with TR. The aim of this review is to provide an overview of the current role of CMR in the assessment and management of patients with TR and its future applications.

## 1. Introduction

Tricuspid regurgitation (TR) is a prevalent valvular heart disease that increases with age. Although often regarded as a bystander in various cardiac and respiratory diseases, severe TR is not benign, as it has a mortality rate of about 40% with medical management [1]. A comprehensive assessment of TR severity and its impact on the right heart is essential for therapeutic management and optimization of patient outcomes. Echocardiography is the first-line imaging technique to evaluate TR, but it is operator-dependent and lacks accuracy with regard to quantifying TR severity and right heart chamber dimensions and function [2]. Cardiovascular magnetic resonance imaging (CMR) has increasingly emerged as an innovative and comprehensive non-invasive cardiac imaging technique. Besides being considered the gold standard for the assessment of cardiac volumes and function, there is a growing role for CMR in the evaluation of valvular heart disease regarding accurate quantitation of valvular lesion severity and risk stratification. When focusing on tricuspid valve disease, CMR can add important insights regarding tricuspid valve anatomy, quantification of TR and assessment of the impact on the right heart. In addition to echocardiography and CMR, tricuspid valve morphology and associated right heart remodeling can also be assessed by cardiac CT. While cardiac CT is increasingly used in patients eligible for transcatheter procedures to obtain detailed anatomical information on the tricuspid valve, right heart and vascular structures [1,2], its overall role in patients with TR remains unclear, and this technique is not mentioned in current guidelines [3,4]. The strengths and weaknesses of echocardiography, CMR and cardiac CT in the assessment of patients with TR are summarized in Table 1.

This review aims to provide an overview of the assessment of TR by CMR, including its added value over routine evaluation by echocardiography, its role according to international guidelines, and its future applications.

## 2. Morphological Analysis of Tricuspid Valve

**Figure 1:**
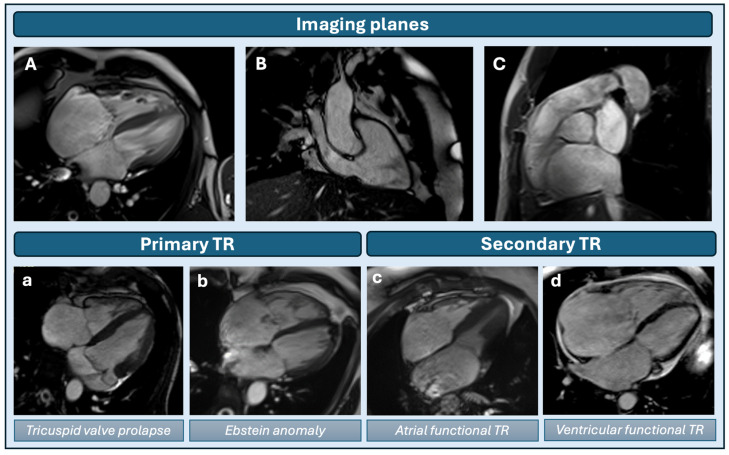
Morphological tricuspid valve analysis and etiology of tricuspid regurgitation. Upper panel: imaging planes to evaluate tricuspid valve morphology. Four-chamber view (panel (**A**)), right ventricular inflow–outflow view (panel (**B**)) and right ventricular outflow tract view (panel (**C**)). Lower panel: examples of primary and secondary tricuspid valve pathology. Panel (**a**): patient with mitral valve prolapse and mitral regurgitation, also tricuspid valve prolapse (orange arrows point at the prolapsing leaflets) with dilated right atrium due to underlying TR. Panel (**b**): patient with Ebstein anomaly (orange arrow points at the apical displaced septal leaflet) and TR (blue arrow points at the TR jet) with dilated right ventricle and right atrium. Panel (**c**): patient with bi-atrial dilatation (dilated LA and RA indicated), normal RV size and functional TR. Panel (**d**): patient with RV infarction, right heart dilatation (dilated RA and RV indicated) and functional TR. LA = left atrium; RA = right atrium; RV = right ventricle; TR = tricuspid regurgitation.

As part of the evaluation of any significant valvular regurgitation, a comprehensive structural assessment of valve morphology is crucial to determine the etiology and guide treatment. CMR allows convenient characterization of tricuspid valve anatomy, including important structural pathology to the leaflets, annulus, papillary muscles or chordae. For detailed anatomical assessment, balanced steady-state free precession (bSSFP) cine images are acquired in different slice planes focused on the tricuspid valve and right ventricle: four-chamber (4CH), right ventricular long axis (RVLA), right ventricular outflow tract (RVOT) and right ventricular inflow and outflow tract (RVIO) views (Figure 1, upper panel) [5]. By acquiring data during multiple cardiac cycles, a dynamic image of tricuspid valve motion can be obtained. Using cross-referencing between short-axis slices (SAX) and other long-axis slices (as prescribed above), all three tricuspid valve leaflets, as well as the papillary muscles, can be identified and evaluated. If any leaflet abnormalities are suspected, an additional stack of continuous thin slices (4 or 5 mm, without gap) should be acquired parallel to the abnormal leaflet [5]. Using these CMR techniques, leaflet thickening, prolapse, tenting, restriction and perforation can be diagnosed. However, due to the limited spatial and temporal resolution, small mobile masses or chordae are more difficult to image.

In addition, CMR allows accurate measurement of tricuspid valve annulus diameters. Normal reference values have been established [6]. In men, a tricuspid annulus of >44 mm (>23 mm/m^2^) measured in 4CH at end-systole or >41 mm (>22 mm/m^2^) at end-diastole is recognized to be dilated. Correspondingly, annular dilatation in women is defined as >39 mm (>23 mm/m^2^) at end-systole or >37 mm (>23 mm/m^2^) at end-diastole [6]. Recently, the concept of tricuspid annulus dysjunction, defined as the distance between the tricuspid annulus and the top of the right ventricular wall, has been investigated using CMR. In a cohort of patients with mitral annulus dysjunction, 50% had a certain degree of tricuspid annulus dysjunction with a mean distance of 5 mm measured at end-systole in 4CH and RVIO views [7].

## 3. Tricuspid Regurgitation Etiology

After morphological analysis of the tricuspid valve, TR etiology can be determined and classified into two subtypes according to the underlying pathophysiology: primary and secondary TR (Figure 1, lower panel). Primary TR can be defined as the presence of any structural valve abnormality, such as congenital disorders, myxomatous degeneration with prolapse, carcinoid syndrome, endocarditis, rheumatic heart disease, etc. In patients with congenital heart disease, like Ebstein anomaly, CMR can provide an accurate evaluation of the complex cardiac anatomy and quantify any possible shunts (Figure 1b). Other causes of primary TR, such as tricuspid valve prolapse (TVP), can also be easily diagnosed with CMR using the following proposed criteria: atrial leaflet displacement of ≥2 mm for the anterior/posterior leaflets and ≥3 mm for the septal leaflet (Figure 1a) [8]. A recent study by Guta et al. [8] showed that TVP is more prevalent in patients with mitral valve prolapse (especially bileaflet prolapse) compared to other causes of primary MR and that prevalence was higher in patients with severe MR compared to mild/moderate MR. Interestingly, patients with TVP had larger tricuspid annuli and larger right ventricular volumes but lower right ventricular ejection fractions. Another growing cause of primary TR is the acquired form induced by implanted cardiac device leads. While transesophageal echocardiography remains the leading non-invasive imaging technique to assess lead-induced TR, it can be safely assessed by CMR as well in many cases, given the increasing number of MR-conditional devices [9,10,11]. Of note, it can be more difficult to evaluate small vegetations in the setting of possible endocarditis due to the lower temporal and spatial resolution of CMR compared to transesophageal echocardiography.

Secondary TR occurs in structural normal valves and typically results from an underlying condition in the right ventricle and/or atrium. Secondary TR is much more prevalent than primary TR. The first subtype, ventricular functional TR, develops from diseases that cause right ventricular (RV) dilatation: pulmonary hypertension, coronary artery disease with RV infarction, left-sided valvular heart disease or left ventricular dysfunction. Significant RV remodeling leads to tethering of the tricuspid valve leaflets and annular dilatation with progression of TR as a consequence [12]. Besides ventricular functional TR, another subtype has emerged as a distinct entity: atrial functional TR (Figure 1c). Due to longstanding atrial fibrillation, progression of TR may occur due to underlying atrial remodeling [13,14,15]. In this subgroup of patients, annular dilatation occurs as a result of significant atrial dilatation, often in the absence of significant RV remodeling [16,17,18]. This leads to a leaflet-to-annulus imbalance and insufficient coaptation without important leaflet tethering, as observed in ventricular functional TR. Interestingly, atrial functional TR can also be present in the absence of atrial fibrillation in patients with heart failure with preserved ejection fraction and bi-atrial dilatation with associated atrial myopathy [19].

## 4. Quantification of Tricuspid Regurgitation

Besides valve morphology, CMR can be used to evaluate TR severity using both qualitative and quantitative methods. First, a gross estimation of the degree of TR severity can be made by visualizing the size of the TR jet on bSSFP cine images, which is represented as signal void due to turbulent flow and/or acceleration. If the TR jet is not clearly seen on bSSFP sequences, spoiled gradient echo with longer repetition and echo times can be used to enhance visualization of the TR jet. Second, CMR can assess TR severity more accurately by quantifying the tricuspid regurgitant volume (TRvol) and fraction (TRF) using indirect or direct methods.

**Figure 2:**
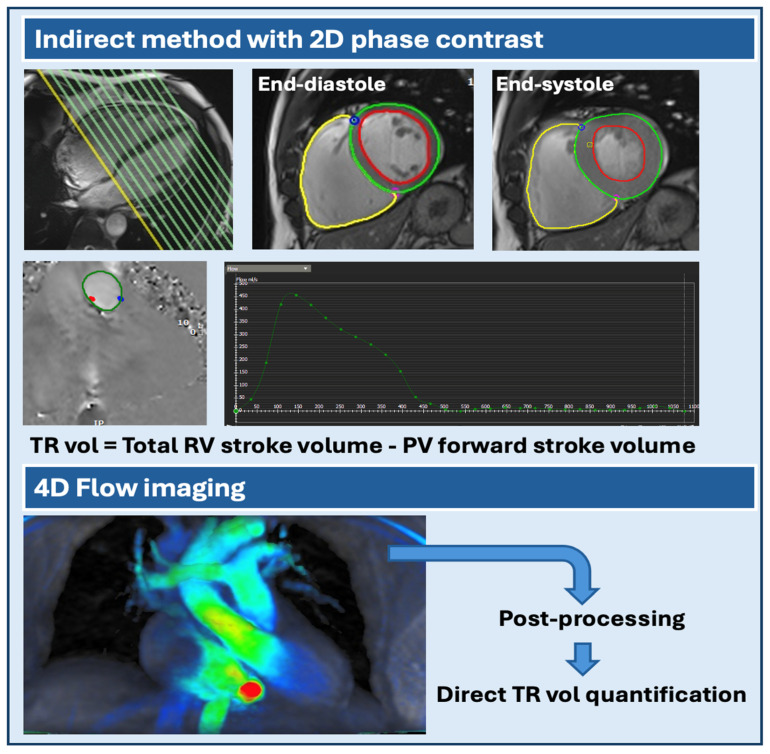
Quantification of tricuspid regurgitation by 2D phase contrast (indirect method) versus 4D flow imaging. Upper panel: Indirect method to quantify TR volume with short-axis stack to calculate RV stroke volume (= RV end-diastolic volume − RV end-systolic volume) and 2D phase contrast to obtain pulmonary valve flow. TR vol = Total RV stroke volume − PV stroke volume. Lower panel: Direct TR volume quantification by 4D flow imaging. Volumes are calculated using post-processing software (Tempus Pixel v32.2). RV = right ventricle; PV = pulmonary valve; TR = tricuspid regurgitation.

Most frequently, TR is quantified indirectly by subtracting pulmonary artery forward flow from the RV stroke volume to calculate TRvol (in the absence of intra-ventricular shunts). Subsequently, TRF can be determined by dividing the TRvol by the RV stroke volume (Figure 2, upper panel). Pulmonary artery forward flow is obtained from a specific CMR sequence called ‘phase contrast flow velocity imaging’. The total flow is calculated by obtaining a slice perpendicular and just superior to the pulmonary valve and by quantifying and integrating the velocity of each pixel and its area during one cardiac cycle on phase velocity maps. The region of interest, in this case, the pulmonary artery, can be outlined on the magnitude image. Phase contrast imaging has been extensively validated and shown to have high intra- and interobserver reproducibility [20,21]. In addition, RV stroke volume can be determined by subtracting the end-systolic from the end-diastolic RV volume. Both volumes are calculated from the biventricular short-axis stack with a slice thickness of 6–8 mm and an interslice gap of 2–4 mm [5]. For each slice, the RV endocardial borders are traced at end-systole and end-diastole. Subsequently, volumes of all slices are combined to produce the total right ventricular volume, in analogy to Simpson’s method [22].

Besides the indirect volumetric method, it is possible to perform direct quantification of tricuspid valve flow using 2D phase contrast flow velocity imaging. However, this method is considered less accurate due to difficulties with the in-plane motion of the tricuspid valve annulus during systole and, in some cases, even the inability to acquire images in a plane perpendicular to the (eccentric) TR jet.

Recently, four-dimensional (4D) flow imaging has emerged as a novel time-resolved three-dimensional (3D) imaging technique that enables visualization and direct quantification of valvular flow and peak velocities (Figure 2, lower panel). Therefore, it overcomes many limitations of indirect quantification with phase contrast flow velocity imaging since the analysis plains can be modified according to the angulation of the valve and the blood flow direction. A standard 4D flow acquisition has a temporal resolution of 30–40 ms and a spatial resolution of less than 3 mm × 3 mm × 3 mm [23]. While a typical 4D flow whole-heart acquisition takes around 5–10 min, several acceleration techniques have been developed to make it more applicable to clinical practice [24,25,26,27].

There are only a limited number of studies that examined the use of 4D flow imaging in patients with TR [28,29,30]. In a cohort of healthy volunteers and patients with TR in the context of ischemic cardiomyopathy, there was a strong agreement between aortic forward flow (as reference) and 4D assessment of tricuspid valve flow [29]. In addition, a good correlation between 2D phase contrast CMR and 4D flow CMR quantification of TR has been observed (direct and indirect methods) [28,29,30]. Even in a cohort of patients with complex RV morphology due to pressure overload or in patients with congenital heart disease, 4D flow has proven to be an effective and reproducible technique [28].

## 5. Analysis of Right Heart Remodeling and Function

As a consequence of severe TR and the associated pressure and volume overload, right ventricular and atrial (RA) remodeling may occur. As the gold standard for quantification of cardiac volumes and the unique advantage of performing tissue characterization, CMR is the ideal non-invasive imaging modality to assess the effects of TR on the right heart. As mentioned previously, RV volumes can be calculated without geometrical assumptions by combining the end-systolic and end-diastolic volumes using cine images of multiple slices covering the RV. In analogy, RA volumes can be calculated if the SAX stack includes extra slices covering the atria. Normal values for RV volumes, RA volumes and RV ejection fraction (RVEF) have been established in multiple cohorts of healthy volunteers [31,32,33]. RV volumes have been shown to decrease with age and tend to be larger in men compared to women, even after correction for body surface area (BSA). Upper limits of normal for RV end-diastolic volumes are, respectively, 123 mL/m^2^ for men and 104 mL/m^2^ for women [31]. Likewise, RA volumes are larger in men, with RA enlargement defined as more than 76 mL/m^2^ in men and 71 mL/m^2^ in women [30]. Furthermore, RVEF can be calculated from CMR-derived RV end-diastolic and end-systolic volumes. Cutoffs of abnormal RVEF have been defined as 42% in men and 47% in women [31]. While RVEF is an important parameter for evaluating global RV systolic function, it cannot assess regional contractility and deformation. In this regard, CMR feature tracking has been developed as a post hoc analysis technique to evaluate myocardial deformation and provide strain parameters from conventional SSFP cine images [34]. The technique has first been optimized for LV analysis, but its assessment of RV strain has been recognized as well [35,36,37,38,39]. While CMR and echocardiography-derived global longitudinal strain values are comparable for the LV, this was not true for the RV, potentially explained by the fact that feature tracking can be challenging in the thin and highly trabeculated RV free wall [40]. In addition to RV volumes and function, RV mass can be easily determined using CMR and is derived from SAX measurements [31].

A unique feature of CMR is the ability to perform tissue characterization with native T1 mapping or late gadolinium enhancement (LGE) after infusion of a Gadolinium-based contrast agent. It has to be mentioned that fibrosis is more challenging to evaluate in the thin RV myocardium compared to the more muscular LV. As a marker for focal fibrosis, LGE can be observed in the setting of (transmural) RV infarction or at the RV insertion points in conditions like pulmonary hypertension [41,42]. Even in patients with primary left-sided heart disease and focal fibrosis on CMR, up to 30% presented with LGE in the RV as well [43]. Native T1 mapping of the RV has been shown to be feasible when performed during systole, and normal values have been defined for 1.5T [44] and 3T [45] scanners. The reproducibility and feasibility of this technique has been investigated as well in patients with pulmonary hypertension [46,47] and congenital heart disease [48]. Moreover, the extracellular volume, which can be calculated from native and post-contrast T1 times, has been investigated as a marker for interstitial fibrosis in the RV as well [47,49].

## 6. Risk Stratification

**Figure 3:**
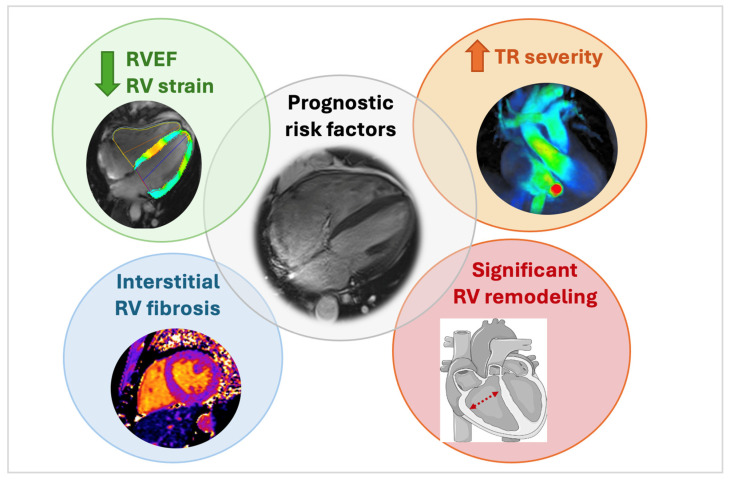
Depiction of risk factors associated with poor outcome in patients with tricuspid regurgitation. Different CMR-derived risk factors in patients with significant tricuspid regurgitation: increasing TR severity, RV remodeling, reduced RVEF & RV strain and markers of fibrosis assessed by tissue characterization (native T1 map). RV = right ventricle; RVEF = right ventricular ejection fraction; TR = tricuspid regurgitation.

When evaluating a patient with any valve lesion, including TR, it is important to assess the risk for associated mortality and morbidity, such as progression to heart failure or need for surgery. A CMR study can provide important insight into this risk stratification, as outcomes are closely related to the volume and pressure overload on the right heart linked to TR (Figure 3).

First, it is essential to correctly assess TR severity and its underlying etiology since both factors are known to affect outcomes and determine the need and timing for intervention. The prognostic role of CMR in this evaluation has been highlighted by the following studies, mainly performed in patients with secondary TR. Zhan et al. [50] showed that increased severity of TR (calculated as TRvol and regurgitant fraction (TRF)) was associated with mortality in patients with functional TR, even after adjustment for other clinical and imaging parameters. A TRvol ≥ 45 mL and TRF ≥ 50% had the greatest risk for excess mortality during follow-up (when medically treated). Wang et al. [51] studied a cohort of patients with moderate–severe or severe isolated TR (21% primary and 79% secondary TR) using both transthoracic echocardiography and CMR. They confirmed the prognostic value of CMR-derived TR volume and TRF, with TRvol ≥ 35 mL and TRF ≥ 30% as optimal thresholds, whereas echocardiographic TR parameters showed no significant association with outcome. In patients with heart failure and secondary TR, CMR-quantified TR severity was significantly associated with a composite endpoint of all-cause mortality and heart failure hospitalization [52].

Second, markers of significant RV remodeling have been associated with poor survival rates. A study by Park et al. demonstrated that impaired RVEF and increased indexed RV end-systolic volume were associated with more post-operative events and cardiovascular death in patients with severe functional TR undergoing tricuspid valve surgery [53]. Similarly, indexed RV mass has been associated with worse prognosis in a cohort of patients with functional TR [54]. In patients undergoing tricuspid valve surgery, postoperative RVEF and decreases in RV volumes are significantly associated with preoperative RV volumes measured by CMR [55]. Moreover, RV strain parameters, which assess longitudinal or circumferential RV contractility, have gained a lot of interest recently. Importantly, survival after transcatheter tricuspid valve repair could be predicted by a worse RV contraction pattern, with RVEF < 45% and diminished global longitudinal and circumferential RV strain [56]. The prognostic role of RV longitudinal strain was further highlighted in a study by Romano et al., who demonstrated increased mortality risk in patients with severe TR and RV strain ≥ −16%. Interestingly, this prognostic effect of RV strain remained significant even after correction for clinical and other imaging parameters [57].

Third, the presence of diffuse interstitial fibrosis, as evaluated by CMR using T1 mapping, has been correlated with adverse RV remodeling in two cohorts of PHT patients. Both increased T1 times at the RV insertion points [58] and increased extracellular volume (ECV) [49] in the RV myocardium were associated with RV dilatation and systolic dysfunction. While these studies display the potential benefit of tissue characterization by T1 mapping for risk prediction of significant TR, more data are needed to verify whether these markers can guide management and thereby improve outcomes.

## 7. Role of CMR in Current Guidelines

As a non-invasive imaging modality, CMR has proven to be safe and useful in many cardiac diseases, including tricuspid valve disease. Yet, the question remains as to what CMR can add to our routine clinical practice and decision-making for patients with significant TR. Current guidelines on valvular heart disease mainly recommend the use of CMR in patients with suboptimal echocardiographic quality or discrepant results. For the assessment of TR specifically, the use of CMR is encouraged to assess right heart size and function [3,4,59]. If the echocardiographic evaluation of TR severity would be inadequate, calculating TRvol using CMR may be helpful [3]. Importantly, CMR-derived cutoffs to define severe TR have not yet been established in the guidelines. The main indications for surgical tricuspid valve intervention are symptomatic patients with severe primary TR and no signs of RV dysfunction or patients with severe primary/secondary TR (or tricuspid annulus dilatation ≥ 40 mm) undergoing left-sided valve surgery [3,4]. Novel transcatheter techniques to treat severe symptomatic secondary TR are currently only considered for inoperable patients, but their role might expand in the future based on the results of ongoing clinical trials [6,60].

## 8. Future Perspectives

With the quickly evolving spectrum of therapeutic options for patients with severe TR, including minimally invasive surgery and transcatheter procedures, the need for accurate pre-procedural evaluation, planning and prediction of outcomes is growing as well. Therefore, more and larger studies are needed that provide data on specific CMR-derived cutoffs of TR severity and RV dysfunction, as well as early markers of adverse outcomes, to select patients who could benefit from intervention [1]. Importantly, these cutoffs and risk markers should be verified in the different types of TR (primary vs. atrial or ventricular secondary TR), as the underlying pathophysiology can vary widely with the need for a tailored therapeutic strategy.

The use of novel CMR sequences like 4D flow will become more rapid and convenient in the future, allowing for routine implementation in clinical practice. More data are needed to verify whether patient outcome is improved if therapeutic management is based on quantification of TR severity by 4D flow instead of routine 2D flow mapping. Regarding tissue characterization, the implementation of novel dark-blood LGE sequences might be beneficial to detect fibrosis in the thin-walled RV, as these techniques improve blood pool-to-scar contrast as compared to conventional LGE imaging [61], but there are currently no data in patients with TR or RV pathology.

Likewise, the use of advanced imaging analysis for functional assessment of the right heart might expand if proven to be useful in clinical practice. Impaired RV and right atrial strain are known to correlate with poor outcomes in patients with TR [62], but data are lacking on whether strain assessment by CMR using feature tracking is more accurate than speckle tracking by echocardiography in this context. Of note, similar to the assessment of mitral valve disease, 4D feature tracking could emerge as an interesting CMR technique to analyze tricuspid valve morphology and dynamics, but this has not yet been studied [63].

The role of CMR as a non-invasive cardiovascular imaging technique will most likely expand in future guidelines since its evaluation of TR severity and RV remodeling has been shown to be more precise and reproducible than routine echocardiography and—importantly—correlates with outcome.

## 9. Conclusions

To conclude, CMR has emerged as a valuable tool in the evaluation of patients with TR, including assessment of valve anatomy, TR severity and its effect on the right heart. Several CMR-derived parameters have diagnostic and prognostic value, highlighting the significance of multi-modality imaging in patients with TR, and can provide valuable information to improve the decision-making process in the management of TR.

## Figures and Tables

**Table 1:** Comparison of tricuspid valve regurgitation assessment using cardiovascular magnetic resonance (CMR) versus echocardiography and cardiac CT.

Arguments	CMR	Echocardiography	Cardiac CT
**PRO**	Gold-standard RV volumes and ejection fraction; highly reproducible Myocardial tissue characterization to detect focal and interstitial RV fibrosis Imaging sections and planes of choice to visualize tricuspid valve leaflets Quantification of TR with 4D flow (direct jet quantification, all valves simultaneously) Evaluation of pulmonary artery size	Superior valve analysis (excellent temporal and spatial resolution, 3D assessment) Hemodynamic assessment: pulmonary artery systolic pressure, central venous pressure, hepatic vein flow	High spatial resolution with 3D analysis of tricuspid valve Analysis of tricuspid valve annular and subannular morphology Planning of vascular access for transcatheter procedures
**CONTRA**	Temporal and spatial resolution inferior to echocardiography Incompatibility with certain devices (e.g., older pacemakers, defibrillators) Flow quantification inaccurate/suboptimal in irregular heart rhythm	Image quality of transthoracic echocardiography may be limited by the patient’s body constitution; alternative transesophageal echocardiography is an invasive procedure Suboptimal evaluation of RV volume and function (incomplete views, high intra- and interobserver variability) Quantification of TR severity not well validated	Radiation exposure No TR quantification

RV = right ventricle, TR = tricuspid regurgitation.
